# Supplementary material for: Role and mechanism of NCAPD3 in promoting malignant behaviors in gastric cancer
Source: Front Pharmacol. 2024 Apr 22;15:1341039. doi: 10.3389/fphar.2024.1341039 (PMC11070777; doi:10.3389/fphar.2024.1341039)
Supplement: Supplementary file 11 [file DataSheet2.ZIP › GSEA/immunologic/my_analysis.Gsea.1599462003817/GSE5503_LIVER_DC_VS_SPLEEN_DC_ACTIVATED_ALLOGENIC_TCELL_DN.html]

Details for gene set GSE5503\_LIVER\_DC\_VS\_SPLEEN\_DC\_ACTIVATED\_ALLOGENIC\_TCELL\_DN[GSEA]

|  || Dataset | filtered\_dataset.sample\_info.cls#WT\_versus\_NCAPD3\_MUT |
| Phenotype | sample\_info.cls#WT\_versus\_NCAPD3\_MUT |
| Upregulated in class | WT |
| GeneSet | GSE5503\_LIVER\_DC\_VS\_SPLEEN\_DC\_ACTIVATED\_ALLOGENIC\_TCELL\_DN |
| Enrichment Score (ES) | 0.45056647 |
| Normalized Enrichment Score (NES) | 1.9040906 |
| Nominal p-value | 0.0017513135 |
| FDR q-value | 0.51234466 |
| FWER p-Value | 1.0 |
Table: GSEA Results Summary

  

Fig 1: Enrichment plot: GSE5503\_LIVER\_DC\_VS\_SPLEEN\_DC\_ACTIVATED\_ALLOGENIC\_TCELL\_DN      
 Profile of the Running ES Score & Positions of GeneSet Members on the Rank Ordered List

  

| SYMBOL | TITLE | RANK IN GENE LIST | RANK METRIC SCORE | RUNNING ES | CORE ENRICHMENT || 1 | 7170 | TPM3 | 24 | 1.018 | 0.0694 | Yes |
| 2 | 23248 | RPRD2 | 33 | 0.977 | 0.1469 | Yes |
| 3 | 92935 | MARS2 | 143 | 0.741 | 0.1316 | Yes |
| 4 | 22834 | ZNF652 | 164 | 0.711 | 0.1777 | Yes |
| 5 | 11231 | SEC63 | 193 | 0.685 | 0.2159 | Yes |
| 6 | 55172 | DNAAF2 | 224 | 0.657 | 0.2502 | Yes |
| 7 | 64794 | DDX31 | 248 | 0.633 | 0.2875 | Yes |
| 8 | 647087 | C7orf73 | 308 | 0.588 | 0.2951 | Yes |
| 9 | 84135 | UTP15 | 330 | 0.576 | 0.3291 | Yes |
| 10 | 54093 | SETD4 | 377 | 0.548 | 0.3426 | Yes |
| 11 | 1387 | CREBBP | 387 | 0.544 | 0.3825 | Yes |
| 12 | 148304 | C1orf74 | 447 | 0.506 | 0.3832 | Yes |
| 13 | 8737 | RIPK1 | 493 | 0.477 | 0.3914 | Yes |
| 14 | 11060 | WWP2 | 503 | 0.471 | 0.4251 | Yes |
| 15 | 23235 | SIK2 | 523 | 0.460 | 0.4506 | Yes |
| 16 | 153562 | MARVELD2 | 732 | 0.359 | 0.3316 | No |
| 17 | 3434 | IFIT1 | 743 | 0.354 | 0.3546 | No |
| 18 | 54206 | ERRFI1 | 849 | -0.229 | 0.2986 | No |
| 19 | 83548 | COG3 | 1211 | -0.588 | 0.0891 | No |
| 20 | 114907 | FBXO32 | 1248 | -0.625 | 0.1165 | No |
Table: GSEA details [plain text format]

  

Fig 2: GSE5503\_LIVER\_DC\_VS\_SPLEEN\_DC\_ACTIVATED\_ALLOGENIC\_TCELL\_DN      
 Blue-Pink O' Gram in the Space of the Analyzed GeneSet

  

Fig 3: GSE5503\_LIVER\_DC\_VS\_SPLEEN\_DC\_ACTIVATED\_ALLOGENIC\_TCELL\_DN: Random ES distribution      
 Gene set null distribution of ES for **GSE5503\_LIVER\_DC\_VS\_SPLEEN\_DC\_ACTIVATED\_ALLOGENIC\_TCELL\_DN**
